# Non-Invasive Device-Mediated Drug Delivery to the Brain across the Blood–Brain Barrier

**DOI:** 10.3390/pharmaceutics16030361

**Published:** 2024-03-05

**Authors:** Toshihiko Tashima, Nicolas Tournier

**Affiliations:** 1 Tashima Laboratories of Arts and Sciences, 1239-5 Toriyama-cho, Kohoku-ku, Yokohama 222-0035, Japan; 2 Laboratoire d’Imagerie Biomédicale Multimodale, BIOMAPS, Université Paris-Saclay, CEA, CNRS, Inserm, Service Hospitalier Frédéric Joliot, 4 Place du Général Leclerc, 91401 Orsay, France

We will be serving as the Guest Editor for this very interesting Special Issue on “Non-Invasive Device-Mediated Drug Delivery to the Brain Across the Blood–Brain Barrier”. It is well-known that the blood–brain barrier (BBB) [1,2], which is substantially composed of tight junctions [3] between the capillary endothelial cells and efflux transporters such as multiple drug resistance 1 (MDR1, P-glycoprotein) [4] at the apical membrane of the capillary endothelial cells, prevents drugs from entering the brain. Accordingly, drug delivery into the brain across the BBB is a challenging task, particularly in central nervous system (CNS) diseases such as Alzheimer’s disease (AD) [5,6] and Parkinson’s disease (PD) [7], as well as brain cancers such as glioma [8]. It is true that drugs in systemic circulation go through intentional membrane disruption or intentional tight junction disruption into the brain across the BBB [9], but bystander harmful compounds can enter the brain together. Moreover, although craniotomy is often conducted for surgical removal or direct drug administration, this process burdens and torments patients. Thus, non-invasive, device-mediated drug delivery across the BBB should be developed to improve patients’ health and quality of life. At present, brain-based drug delivery systems that utilize biological transport machineries such as carrier-mediated transport, receptor-mediated transcytosis, lipid-raft-mediated transcytosis, or macropinocytosis at the BBB have been extensively investigated [10]. This Special Issue aims to share the recent progress and trends in this field.

The delivery of drugs across the cell membrane is achieved using vectors such as monoclonal antibodies (mAbs) [11], cell-penetrating peptides (CPPs) [12], or tumor-homing peptides (THPs) [13]. It is suggested that negatively charged heparan sulfate chains branching from proteoglycan (HSPG) on the cell surface induce receptor-mediated endocytosis as a receptor for cationic CPPs [14]. RGD peptides (Arg-Gly-Asp), as representative THPs, specifically target cancer cells by binding to ανβ3 and ανβ5 integrins [15]. NGR peptides (Asn-Gly-Arg) bind to the receptor aminopeptidase N [16]. Sarfaraz K. Niazi outlines current and future approaches to enhance BBB penetration to treat multiple brain diseases using such delivery technology [17]. Nikesh Gupta et al. present CPPs- or THPs-mediated delivery into the cells [18]. The mechanisms of CPP internalization, involving endocytosis and direct translocation, are widely recognized. The detailed mechanisms of CPPs, specifically regarding membrane internalization and endosomal escape, are accurately described. Both CPPs with cargo and THPs with cargo were endocytosed in the capillary endothelial cells at the BBB. Moreover, Maarten Dewilde et al. introduce mAbs-mediated transcytosis into the brain across the BBB, using nanobodies against the transferrin receptor (TfR) [19]. They developed an anti-TfR nanobody-anti-BACE1 mAb bispecific conjugate. Intravenously administered bispecific conjugates lowered Aβ1–40 levels in plasma in an in vivo assay using hAPI KI mice, in which the mouse TfR apical domain was replaced by the human sequence. These bispecific conjugates entered the brain across the BBB via TfR-mediated transcytosis and inhibited BACE1 in the brain/cerebrospinal fluid (CSF). Currently, nanobodies [20] are attracting considerable attention due to their compact size and high specificity. Furthermore, Izcargo^®^ (pabinafusp alfa), clinically launched in Japan in May, 2021, for the treatment of all forms of MPS II, enters the brain across the BBB via receptor-mediated transcytosis using TfR. The brain drug delivery technology J-Brain Cargo^®^ is utilized in this drug, composed of the conjugate between anti-TfR monoclonal antibody and human iduronate-2-sulfatase [21]. Thus, drug delivery into the brain via TfR-mediated transcytosis could be a promising strategy. Moreover, it is reported that the clustering of ligand-receptor complexes derived from TfRs enhances endocytosis [22,23]. Generally, clustering induces endocytosis [10,24,25].

In addition, carrier-mediated transport into the brain might be conducted for low-molecular-weight *N*-containing drugs using the proton-coupled organic cation antiporter [26]. Most CNS drugs have structurally incorporated *N*-containing groups into their molecules. It is well-known that certain pharmaceutical agents, such as CNS drugs and antihistamine drugs, can penetrate the brain through the BBB. It is suggested that certain cation transporters facilitate the transport of *N*-containing drugs across the BBB. Memantine for AD is positively charged under physiological pH and, therefore, cannot cross the membrane via passive diffusion. Indeed, memantine with an *N*-containing group is transported into cells via carrier-mediated transport [27]. In general, compounds are divided into three categories, that is, low-molecular-weight compounds (molecular weight (MW) < approx. 500), high-molecular-weight compounds (MW > approx. 3000), and middle-molecular-weight compounds (MW approx. 500–approx. 3000) [10]. High-molecular-weight compounds such as monoclonal antibodies cannot penetrate through the pores of solute carrier transporters, while hydrophilic low-molecular-weight compounds are facilitated by solute carrier transporters. Hydrophobic low-molecular-weight compounds cross the cell membrane via passive diffusion, although they are substrates of MDR1. Thus, the transport strategies, including the transcellular pathway, such as passive diffusion, carrier-mediated transport, or receptor-mediated transcytosis, and the paracellular pathway such as transport through disrupted tight junctions, depend on the molecular size and hydrophobicity, based on the machinery systems regulated by structuralism [28,29].

Furthermore, nanodelivery systems utilizing nanoparticles are innovative tools for delivering cargo drugs to target sites, particularly the brain or cancer tissues [30,31,32]. Various surface modifications can easily be made to nanoparticles. Encapsulated substances are protected from enzymatic degradation and are not prone to off-target side effects. David J. Daniels et al. provide nanoparticle strategies for delivering drugs into the brain across the BBB, particularly for the treatment of brain tumors via receptor-mediated transcytosis or other internalization mechanisms. Various types of nanoparticles are engineered to enhance targeted delivery into the brain. Nanoparticle clearance and blood circulation time are also crucial to avoid serious side effects [33]. Lars Esser, Nicolas H. Voelcker et al. synthesized porous silicon nanoparticles (PSiNPs) covered with transferrin (average size of 203 and 420 nm). The association of hCMEC/D3 with PSiNPs was enhanced as transferrin content increased from 0 nmol/mg to 3.8 nmol/mg. It was clarified that an intermediate transferrin surface density showed the highest BBB transport. The smaller PSiNPs consistently exhibited higher BBB penetration potential than the larger PSiNPs via receptor-mediated transcytosis [34]. These findings are valuable for nanoparticle design. Nanoparticles should be developed using biocompatible and biodegradable polymers [35]. Poly(lactic acid) (PLA) and poly(lactic-co-glycolic acid) (PLGA) are often utilized [36]. The most common form endocytosis is clathrin-mediated endocytosis [37], inducing endosomes (85–150 nm in diameter) [10], although there are various types of endocytosis [38]. Therefore, the size of the internalized nanoparticles should be within the range of these endosomes. Interestingly, the pH in endosomes gradually decreases from the early endosome (pH approx. 6.5) to the late endosome (pH approx. 5.5), and finally becomes the lysosome (pH approx. 4.5) due to the vacuolar H^+^-ATPase proton pumps in the degradation pathway [39]. Such acidification can be utilized for cargo release through the leakage of pH-sensitive linkers. The released cargos might penetrate the membrane of endosomes, leading to endosomal escape, or may penetrate the membrane of lysosomes, leading to lysosomal escape, via passive diffusion. On the other hand, endosomes or lysosomes burst through the proton sponge effect in the case of amine-rich carriers while acidification proceeds [40].

Broadly speaking, nose-to-brain drug delivery is a strategy to deliver drugs into the brain without crossing the BBB [41,42]. Strictly speaking, this pathway does not involve the BBB. Vivek Trivedi et al. provide an overview of the current state of intranasal formulation development for nose-to-brain drug delivery and summarize the biologics that are currently undergoing clinical trial [43]. Intranasally administered substances can be transported across the olfactory epithelium and subsequently move into the brain through the olfactory nerve or trigeminal nerve. Murali Monohar Pandey et al. developed rotigotine-loaded lecithin-chitosan nanoparticles (RTG-LCNP) for the treatment of PD [44]. RTG-LCNP showed a 9.66-fold increase in the amount permeated compared to pure drug suspension in an ex vivo nasal permeation study using male Wistar rats. On the other hand, mesenchymal stem cells (MSCs) [45] administered through intravenous or intracarotid routes can be utilized as a drug carrier, homing to the target sites, although they are often clinically used for regenerative medicine due to their differentiation potential [46]. Toshihiko Tashima proposes MSC-based drug delivery into the brain across the BBB [47]. The substances delivered by MSCs are divided into artificially included materials in advance, such as low-molecular-weight compounds including doxorubicin, and the expected protein expression products of genetic modification, such as interleukins.

Screening methods to analyze drug permeability across the BBB are important for CNS drug development [48]. Susan Hawthorne et al. developed a viable method for the high-throughput screening of CNS drugs using a novel transwell human BBB model. Fitc-dextran-encapsulated PLGA nanoparticles covered with DAS peptide were transported via receptor-mediated transcytosis that is 14-fold greater than Fitc-dextran-encapsulated PLGA nanoparticles in this assay system [49]. A variety of nanoparticles can be effectively evaluated through this system. Marie-Anne Estève demonstrates the transportation of imaging compounds into the brain through transient FUS-mediated BBB opening performed on healthy animals [50]. CNS imaging is increasingly recognized for its vital role in preventive medicine for neurodegenerative diseases such as AD in an aging society [51]. Tau imaging [52,53] and Aβ imaging [54,55] will play an important role in confirming the progress of AD for early intervention [56] because the number of AD patients is expected to increase in the future [57]. It is likely difficult to cure AD once the symptoms have progressed to a certain extent. The social losses, such as costs and the burden of nursing care due to AD, are immeasurable. Recently, several anti-Aβ monoclonal antibodies, such as aducanumab [58] and lecanemab [59], have been clinically approved. Furthermore, donanemab finished a phase 3 clinical trial with favorable results for early AD in 2023 [60]. The development of drugs that can provide a fundamental treatment is good news for AD patients. We hope that this Special Issue will contribute to the creation of innovative medicines.

Overall, the articles in this Special Issue outline non-invasive device-mediated brain drug delivery across the BBB and will contribute to the development of this field. We would like to express our gratitude to all the authors of this Special Issue for their outstanding contributions. Moreover, we extend our thanks to the Assistant Editors, for their valuable assistance.
